# Circular RNA circ_ARHGEF28 inhibits MST1/2 dimerization to suppress Hippo pathway to induce cisplatin resistance in ovarian cancer

**DOI:** 10.1186/s12935-024-03451-w

**Published:** 2024-07-21

**Authors:** Ruilin Lei, Yun Long, Qingjian Li, Qingsheng Xie, Xiaoting Ling, Meiqing Xie, Hui Zhou, Bingzhong Zhang

**Affiliations:** 1 Guangdong Provincial Key Laboratory of Malignant Tumor Epigenetics and Gene Regulation, Guangzhou, 510120 People’s Republic of China; 2 Department of Gynecology and Obstetrics, Sun Yat-Sen Memorial Hospital, Sun Yat-Sen University, 107 Yanjiang West Road, Guangzhou, 510120 People’s Republic of China; 3 Department of Oncology, Sun Yat-Sen Memorial Hospital, Sun Yat-Sen University, Guangzhou, 510120 People’s Republic of China

## Abstract

**Background:**

Cisplatin is integral to ovarian cancer treatment, yet resistance to this drug often results in adverse patient outcomes. The association of circular RNA (circRNA) with cisplatin resistance in ovarian cancer has been observed, but the mechanisms governing this relationship require further elucidation.

**Methods:**

High-throughput sequencing was utilized to profile circRNA expression in cisplatin-resistant ovarian cancer cells. Gain-and-loss-of-function experiments assessed the impact on cisplatin sensitivity, both in vitro and in vivo. Fluorescence in situ hybridization was conducted to determine the cellular distribution of circRNAs, and RNA pulldown and immunoprecipitation experiments were performed to identify associated binding proteins.

**Results:**

The study revealed that circ_ARHGEF28 is overexpressed in certain cisplatin-resistant ovarian cancer tissues and cell lines, and is associated with reduced progression-free survival in patients. It was observed that circ_ARHGEF28 contributes to cisplatin resistance in ovarian cancer models, both in vitro and in vivo. Importantly, circ_ARHGEF28 was found to interact directly with MST1/2, inhibiting the SARAH coiled-coil binding domains and consequently deactivating the Hippo pathway.

**Conclusion:**

This investigation identifies circ_ARHGEF28 as a novel circRNA that contributes to cisplatin resistance in ovarian cancer by suppressing the Hippo pathway. Therapeutic strategies targeting circ_ARHGEF28 may offer a potential avenue to mitigate cisplatin resistance in ovarian cancer treatment.

**Supplementary Information:**

The online version contains supplementary material available at 10.1186/s12935-024-03451-w.

## Introduction

Globally, ovarian cancer ranks as a leading cause of cancer-related death among women [1, 2]. While anti-angiogenesis agents and PARP inhibitors have been integrated into ovarian cancer treatment strategies, cisplatin remains a cornerstone of therapy [3]. However, the development of resistance to cisplatin poses a significant hurdle [4], often leading to treatment failure [5]. Understanding the underlying mechanisms of cisplatin resistance in ovarian cancer is therefore vital.

Circular RNAs (circRNAs), a class of endogenous non-coding RNAs, are characterized by their widespread distribution, diverse functions, structural stability, tissue-specific expression, and evolutionary conservation [6, 7]. These RNAs, predominantly found in the cytoplasm, lack the poly(A) tails typical of 5’ and 3’ ends [8, 9]. Recent research has highlighted the differential expression of circRNAs in tumor-associated tissues and cells, noting their association with drug resistance in various malignancies, including nasopharyngeal carcinoma [10], lung cancer [11], breast cancer [12], and colorectal cancer [13]. Notably, circRNAs have been implicated in modulating tumor resistance to chemotherapy [14]. For instance, circUBAP2 has been shown to contribute to cisplatin resistance in triple-negative breast cancer by binding to miR-300 and engaging the PI3K/AKT/mTOR signaling pathway [15]. While several circRNAs have been reported to facilitate chemoresistance in ovarian cancer, primarily through miRNA sponging, their detailed mechanisms remain inadequately understood [16, 17].

The Hippo pathway, an extensive network of proteins, regulates tissue and organ regeneration during development and influences cancer biology, including treatment resistance [18, 19]. Central to this pathway are pairs of serine/threonine kinases - MST1 and MST2 - along with large tumor suppressor kinases LATS1 and LATS2 [20, 21]. Dimerization of MST1/MST2 activates LATS1 and LATS2, which in turn phosphorylates the oncoproteins YAP and TAZ [22]. This phosphorylation sequesters YAP and TAZ in the cytoplasm, leading to their ubiquitination-induced proteolysis [23].

It has been observed that the Hippo pathway interacts with various non-coding RNAs, including microRNAs, long non-coding RNAs, and circular RNAs, mediating a range of biological processes [7, 24, 25]. In this study, a novel circRNA, circ_ARHGEF28, was identified as a mediator of cisplatin resistance in ovarian cancer. This RNA impedes the interaction within the Hippo pathway by blocking the binding domains of MST1/2, thereby deactivating the pathway and inducing resistance to cisplatin. Knockdown of circ_ARHGEF28 in vitro and in vivo was found to reverse this resistance.

This investigation seeks to elucidate the potential mechanism by which circ_ARHGEF28 mediates cisplatin resistance in ovarian cancer, aiming to provide a theoretical foundation for understanding the pathogenesis of this resistance.

## Materials and methods

### Patients and tissue samples

For the analysis of circ_ARHGEF28 expression in cisplatin-resistant and sensitive ovarian cancer, samples were collected from 150 female patients at Sun Yat-Sen Memorial Hospital, Sun Yat-Sen University, between September 2008 and May 2021. This study was conducted in accordance with the STARD reporting guidelines and the Helsinki Declaration. Informed consent was obtained from all donors, adhering to the International Ethical Guidelines for Biomedical Research Involving Human Subjects (CIOMS). This retrospective study, involving identified data, received approval from the Institutional Review Board (IRB) of Sun Yat-Sen Memorial Hospital [IRB no. SYSKY-2023-114-01], with a waiver for written consent.

### Cells and cell culture

A2780 cells, sourced from the American Type Culture Collection (ATCC), were cultured in DMEM supplemented with 10% FBS, following standard protocols. Cisplatin-resistant A2780 cells (A2780R) were developed through treatment with 10 nmol cisplatin until cell death rates fell below 5%. A2780R cells were maintained in DMEM with 10% FBS, mirroring the conditions for parental A2780 cells.

### RNA preparation and high-throughput sequencing analysis

Total RNA from A2780, cisplatin-resistant A2780R, and ovarian cancer samples (both cisplatin-resistant and sensitive) was extracted using Trizol reagent. For RNA sample preparations, 3 µg of RNA per sample was used. Ribosomal RNA was removed using the Epicentre Ribo-Zero™ Gold Kit (Human/Mouse/Rat) (Epicentre, USA). Following the manufacturer’s recommendations, sequencing libraries were generated using the NEBNext^®^ Ultra™ Directional RNA Library Prep Kit for Illumina (NEB, Ipswich, USA). Library construction entailed the following steps: ribosomal RNA removal, RNA fragmentation under elevated temperatures, and synthesis of the first cDNA strand using random hexamer primers with RNA fragments as templates. The second strand of cDNA was synthesized using a mix of buffer, dNTPs, DNA polymerase I, and RNase R. After purification with QiaQuick PCR kits and EB buffer elution, library fragments underwent end repair, poly(A) addition, and adapter ligation. cDNA fragments of approximately 300 bp were selected through agarose gel electrophoresis, followed by UNG enzyme digestion of the second cDNA strand. PCR amplification was conducted, and the final library was retrieved through agarose gel electrophoresis.

RNA sequencing was performed by OE Biotech Co., Ltd. (Shanghai, China).

### siRNA / shRNA and constructs

Two siRNA/shRNA duplexes, produced by Guangzhou Nuohao Biotechnology Co., Ltd., were utilized as detailed in Supplemental Table 3. The pLKO-Tet-On “all-in-one” plasmid facilitated inducible expression of shcirc_ARHGEF28 and sh-control. For ectopic expression in cells, MST1 and MST2 (full-length and truncated versions), along with circ_ARHGEF28, were cloned into the siRNA/shRNA or pCDNA3.1 plasmid. Transfections were performed using Lipofectamine 3000 (Invitrogen).

### Quantitative RT-PCR

1 µg total RNA, extracted from cultured cells following standard protocols, was reverse transcribed into cDNA using the Superscript First-Strand cDNA Synthesis Kit (18080-051, Invitrogen, Carlsbad, CA). Quantitative RT-PCR was conducted using the SYBR Premix Ex Taq II kit (DRR081A, TAKARA, Otsu, Shiga, Japan) on a Light Cycler 480 System (Roche, Basel, Switzerland).

### Colony formation assay

A total of 1,000 cells were seeded into 6-well plates and cultured for 14 days. Post fixation with 4% formaldehyde for 15 min, colonies were stained with 1% crystal violet.

### IC_50_ and MTS cell viability assay

For each assay, 2,000 cells were plated in 96-well plates and treated with varying concentrations of cisplatin. At designated time points, cells were incubated with a sterile MTS mix (1:9 in culture medium) for 2 h at 37 °C in darkness. Absorbance was measured at 490 nm.

### BaseScope and fluorescence in situ hybridization (FISH)

BaseScope (BaseScope™ Reagent Kit v2-RED, Advanced Cell Diagnostics China, #323,900) was employed for in situ detection of circ_ARHGEF28 expression in ovarian tumor samples. Deparaffinized paraffin-embedded samples were treated with 1×BaseScope solution (Target Retrieval) and hybridized with probes in a humidified box at 40℃ for 2 h. Hybridization was followed by sequential incubation with BaseScope v2 AMP 1 to 8 and washing with RNAscope wash buffer. Signals were detected using BaseScope™ Fast RED and counterstained with hematoxylin. Positivity was identified as red dots or clusters.

The BaseScope signal was quantified by combining the percentage of positive tumor cells with staining intensity, graded as 0 (no staining), 1 (weak, red dot), 2 (moderate, red dot to cluster), or 3 (strong, red cluster). An H-score was calculated using the formula: [1 × (% cells 1+) + 2 × (% cells 2+) + 3 × (% cells 3+)]. This method assessed circ_ARHGEF28 expression in ovarian cancer samples, with the median value defining circ_ARHGEF28-high and circ_ARHGEF28-low expression.

For FISH, standard protocols were followed using a probe targeting circ_ARHGEF28 (RiboBio Co, Ltd, Guangzhou, China). Confocal microscopy involved overnight incubation with fluorescence-conjugated anti-DIG antibodies (Roche) at 4 °C, followed by DAPI counterstaining and imaging with a laser scanning confocal microscope (A1, Nikon).

### Immunoblotting

Cell lysates were prepared using RIPA lysis buffer (Beotime, China) supplemented with protease and phosphatase inhibitors (Life Technologies, USA). Proteins were separated by 8% SDS-PAGE and transferred onto PVDF membranes (Bio-Rad, USA). After blocking with 5% non-fat milk in 0.1% TBST buffer overnight at 4 °C, the membranes were incubated with primary antibodies (dilutions 1:500-1:1000, as listed in Supplemental Table 4). The protein-antibody complexes were detected using HRP-conjugated secondary antibodies and enhanced chemiluminescence. The Image J was used for statistical analysis of the result.

### RNA immunoprecipitation (RIP)

RIP assays were conducted using the Magna RIP RNA Binding Protein Immunoprecipitation Kit (Millipore, MA, USA), following the manufacturer’s guidelines. Cell extracts were prepared in lysis buffer containing protease and RNase inhibitors, incubated on ice for 5 min and centrifuged at 10,000 g at 4 °C for 10 min. Magnetic beads were preincubated with 5 µg of IP-grade antibody for 30 min at room temperature with rotation. The supernatant was then added to the bead-antibody complexes in immunoprecipitation buffer and incubated overnight at 4 °C. RNA was subsequently purified and quantified via qRT-PCR. Input controls and normal rabbit IgG controls were included to confirm the specificity of RNA-protein binding.

### Ubiquitination assay

Cells were treated with MG132 (20 nM) in DMEM culture medium for 4 h before lysate collection. Lysates were immunoprecipitated with specific antibodies on protein A/G beads (Life Technologies) overnight at 4 °C with rotation, then boiled in SDS buffer. The eluted proteins were detected by immunoblotting.

### Circular RNA pull-down assay

Circular RNA pull-down was executed using biotinylated circ_ARHGEF28 (sense) and a negative control (antisense) probes provided by Guangzhou RiboBio Co, Ltd. Initially, cells were lysed in IP lysis buffer at 4 °C for 30 min. Cell supernatants were incubated with circ_ARHGEF28 and control biotinylated probes overnight at 4 °C. The mixtures were then co-incubated with streptavidin magnetic beads at room temperature for 1 h. Following SDS-PAGE separation, proteins were visualized via silver staining. Mass spectrometry analysis was conducted to identify potential binding proteins of circ_ARHGEF28.

### Co-immunoprecipitation (Co-IP) assay

Cell lysates from A2780R were immunoprecipitated with specified antibodies on protein A/G beads (Life Technologies) overnight at 4 °C with rotation, followed by boiling in SDS buffer. The eluted proteins were analyzed using immunoblotting techniques.

### Animal experiments

All animal studies were performed in compliance with the guidelines of the Institutional Animal Care and Use Committee at Sun Yat-Sen University Medical School. Mice were sourced from Beijing Vital River Laboratory Animal Technology Co., Ltd. Six-week-old female nude mice (*n* = 5 per group) were subcutaneously inoculated with indicated cells. Upon reaching an average tumor volume of approximately 150 mm^3^, the mice were administered doxycycline (DOX) to induce DOX-inducible shRNA-circ_ARHGEF28 expression, followed by cisplatin treatment (4 mg/kg intraperitoneally) daily for one week.

### Statistics

In vitro, experimental data were presented as the mean ± standard deviation (S.D.) derived from three independent experiments. Statistical analyses were conducted using the SPSS 16.0 software package (SPSS, Chicago, IL, USA). The Student’s t-test and one-way ANOVA were applied to assess differences in cell viability, colony formation, apoptosis rates, and tumor volumes under various treatment conditions. The Chi-square test was utilized to examine the association between circ_ARHGEF28 expression and clinicopathological characteristics. Survival outcomes, including overall survival (OS) and progression-free survival (PFS), were compared across different patient groups using Kaplan-Meier curves and log-rank tests. Statistical significance was denoted as follows: *P < 0.05, **P < 0.01, and ***P < 0.001.

## Results

### Association of circ_ARHGEF28 overexpression with cisplatin resistance and poor survival in ovarian cancer

**Fig. 1:**
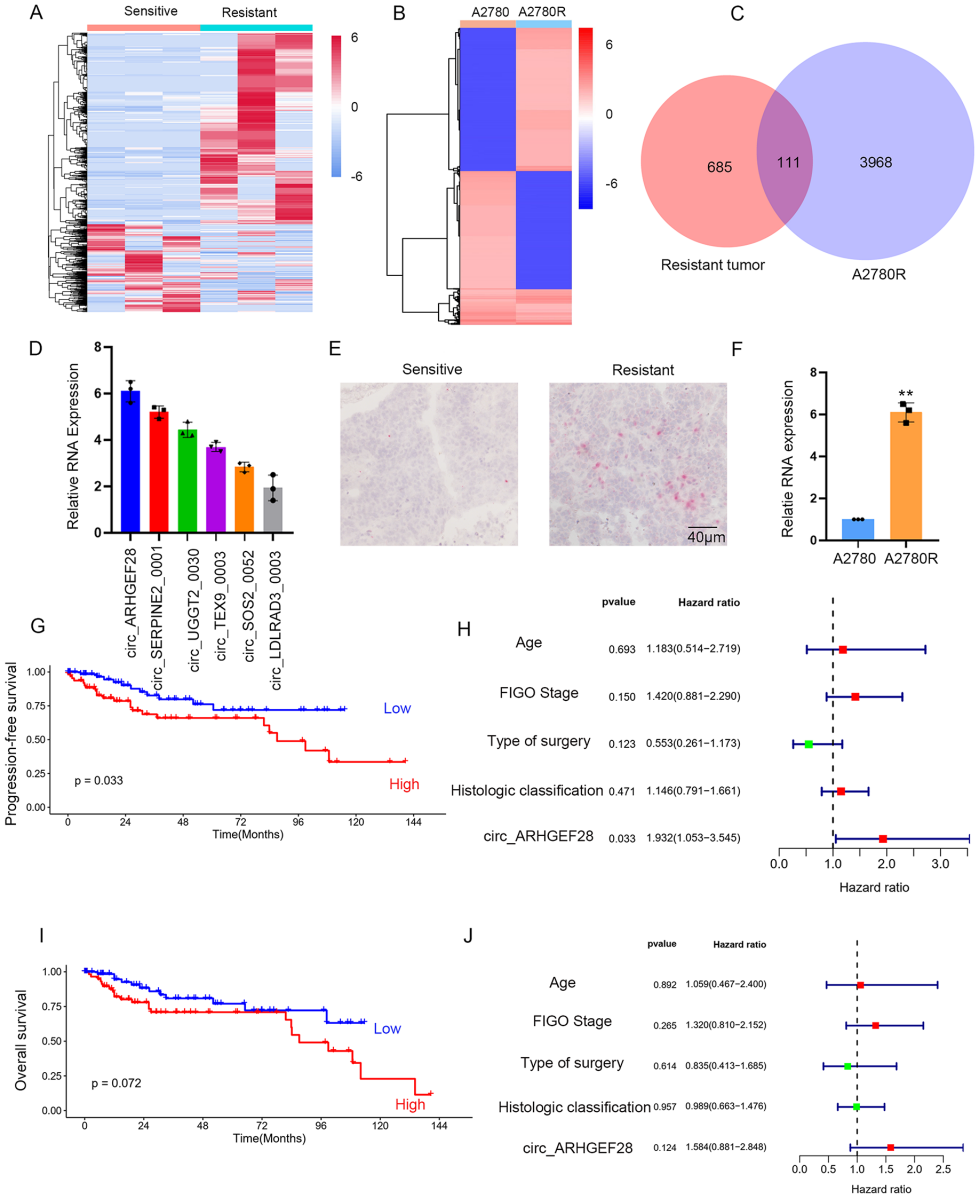
Association of circ_ARHGEF28 Overexpression with Cisplatin Resistance and Poor Survival in Ovarian Cancer. **A**-**B** The heatmaps of circRNAs that were upregulated or downregulated over 6 folds in cisplatin-resistant ovarian cancer tissues (*n* = 3) compared with cisplatin-sensitive ovarian cancer tissues (*n* = 3) of adjuvant chemotherapy (**A**) and circRNAs that were upregulated or downregulated at least 6 folds in cisplatin resistant ovarian cancer A2780R cells comparing to that in parental A2780 cells(**B**). Expression levels were shown as log2 transformed intensity relative to the mean value of all samples. **C**. The number of overlapping circRNAs that were upregulated among cisplatin-resistant ovarian cancer samples and A2780R in **A** and **B**. **D**. The expression of circ_ARHGEF28 was most upregulated among the six upregulated circRNAs in A2780R. **E**. Representative in situ hybridization (ISH) images of circ_ARHGEF28 expression in the cisplatin resistant ovarian cancer samples and cisplatin sensitive ovarian cancer samples. **F**. Expression of circ_ARHGEF28 was upregulated in A2780R cells compared to parental A2780 cells. ** means *p* < 0.01. **G**. Higher levels of circ_ARHGEF28 in ovarian cancer were significantly associated with poor progression-free survival (PFS). **H**. Multivariate Cox regression analysis of multiple factors to progression-free survival (PFS). **I**. Higher levels of circ_ARHGEF28 in ovarian cancer were associated with overall survival (OS), but not significantly. **J**. Multivariate Cox regression analysis of multiple factors to overall survival (OS)

This study aimed to identify crucial circRNAs driving cisplatin resistance in ovarian cancer. To this end, cisplatin-resistant tumor tissues from three patients with no response or relapse within six months of cisplatin-based chemotherapy, and cisplatin-sensitive tissues from patients relapsing beyond six months or remaining relapse-free post-treatment, were analyzed. High-throughput sequencing was employed to compare their circRNA expression profiles (Fig. 1A). Additionally, to delve into the molecular mechanisms behind cisplatin resistance, a resistant ovarian cancer cell line, A2780R, was developed and its circRNA profile contrasted with that of the parental A2780 line (Fig. 1B). Notably, totally 111 circRNAs were upregulated in both resistant tumor samples and A2780R cells (Fig. 1C). Their elevated expression in A2780R cells was confirmed by qRT-PCR compared to A2780 cells. Among these, circ_ARHGEF28 was significantly overexpressed (Fig. 1D). BaseScope analysis of surgically resected cisplatin-resistant and sensitive tumor tissues revealed a higher expression of circ_ARHGEF28 in resistant samples (Fig. 1E), with a six-fold increase in A2780R compared to A2780 as shown by qPCR (Fig. 1F).

In a further evaluation of circ_ARHGEF28’s role in ovarian cancer survival, BaseScope analysis was performed on 150 ovarian cancer tissue samples (Table S1). High expression of circ_ARHGEF28 correlated with significantly worse progression-free survival (PFS) (Fig. 1G), though not overall survival (OS) (Fig. 1I), in patients treated with cisplatin-based chemotherapy (Fig. 1G-H). Moreover, multivariate Cox regression analysis identified circ_ARHGEF28 as an independent prognostic factor for PFS (*p* = 0.033) (Fig. 1H), but not for OS (Fig. 1J). These findings indicate a strong association between elevated circ_ARHGEF28 levels and both cisplatin resistance and reduced PFS in ovarian cancer.

### Characterization and cellular localization of circ_ARHGEF28

**Fig. 2:**
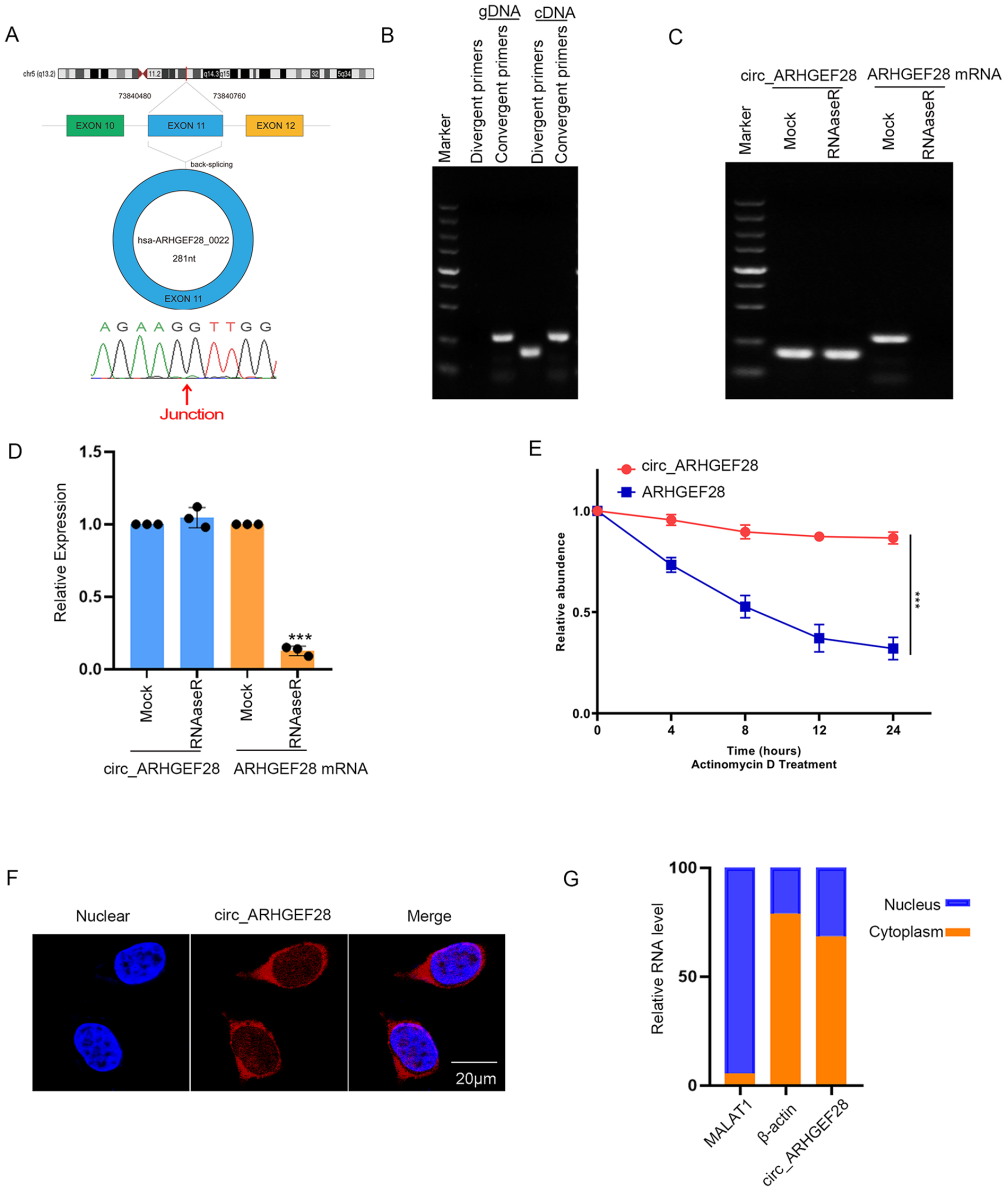
Characterization and Cellular Localization of circ_ARHGEF28. **A**. The annotated region in ARHGEF28 gene for the formation of circ_ARHGEF28 was shown. **B** The circ_ARHGEF28 and linear transcript of ARHGEF28 were detected by divergent and convergent primers in cDNA and gDNA of A2780R cells using PCR. **C**-**D**. The stability of circ_ARHGEF28 and ARHGEF28 mRNA was detected by RNase R degradation assay. The result of qPCR was obtained over three independent experiments. *** means *p* < 0.001. **E**. The stability of circ_ARHGEF28 and ARHGEF28 mRNA was detected by actinomycin D degradation assay. The result of qPCR was obtained over three independent experiments. *** means *p* < 0.001. **F**. Cellular localization of circ_ARHGEF28 in A2780R cells was detected by immunofluorescence. **G**. Relative RNA levels in nucleus and cytoplasm of MALAT1, β-actin, and circ_ARHGEF28 in A2780R cells. The circ_ARHGEF28 is mainly located in cytoplasm as indicated by nucleus/cytosol Fractionation assay

Circular RNAs, by virtue of lacking 5’ or 3’ ends, are inherently resistant to RNase R, an endonuclease that degrades linear RNAs with short 3’ tails but spares lariat or circular forms. This resistance confers greater stability to circular RNAs. Circ_ARHGEF28, derived from exon 11 of the ARHGEF28 gene and spanning 281 nucleotides (nt), exemplifies this stability (Fig. 2A). To confirm its circular structure, divergent and convergent primers were employed to amplify circ_ARHGEF28 and linear ARHGEF28, respectively. Notably, circ_ARHGEF28 was only amplified by divergent primers in cDNA, not in gDNA (Fig. 2B). Additionally, circ_ARHGEF28 exhibited greater stability than ARHGEF28 mRNA post-RNase R treatment (Fig. 2C-D). This enhanced stability was further validated by treating with actinomycin D, demonstrating the circular nature of circ_ARHGEF28 (Fig. 2E).

The cellular localization of circ_ARHGEF28 was determined using fluorescence in situ hybridization (FISH). The results revealed that circ_ARHGEF28 predominantly resides in the cytoplasm (Fig. 2F). This finding was corroborated by nucleus/cytoplasm fractionation, showing that approximately 70% of circ_ARHGEF28 was localized in the cytoplasm of A2780R cells (Fig. 2G).

### The circ_ARHGEF28 promotes cisplatin resistance in ovarian cancer in Vitro and in vivo

**Fig. 3:**
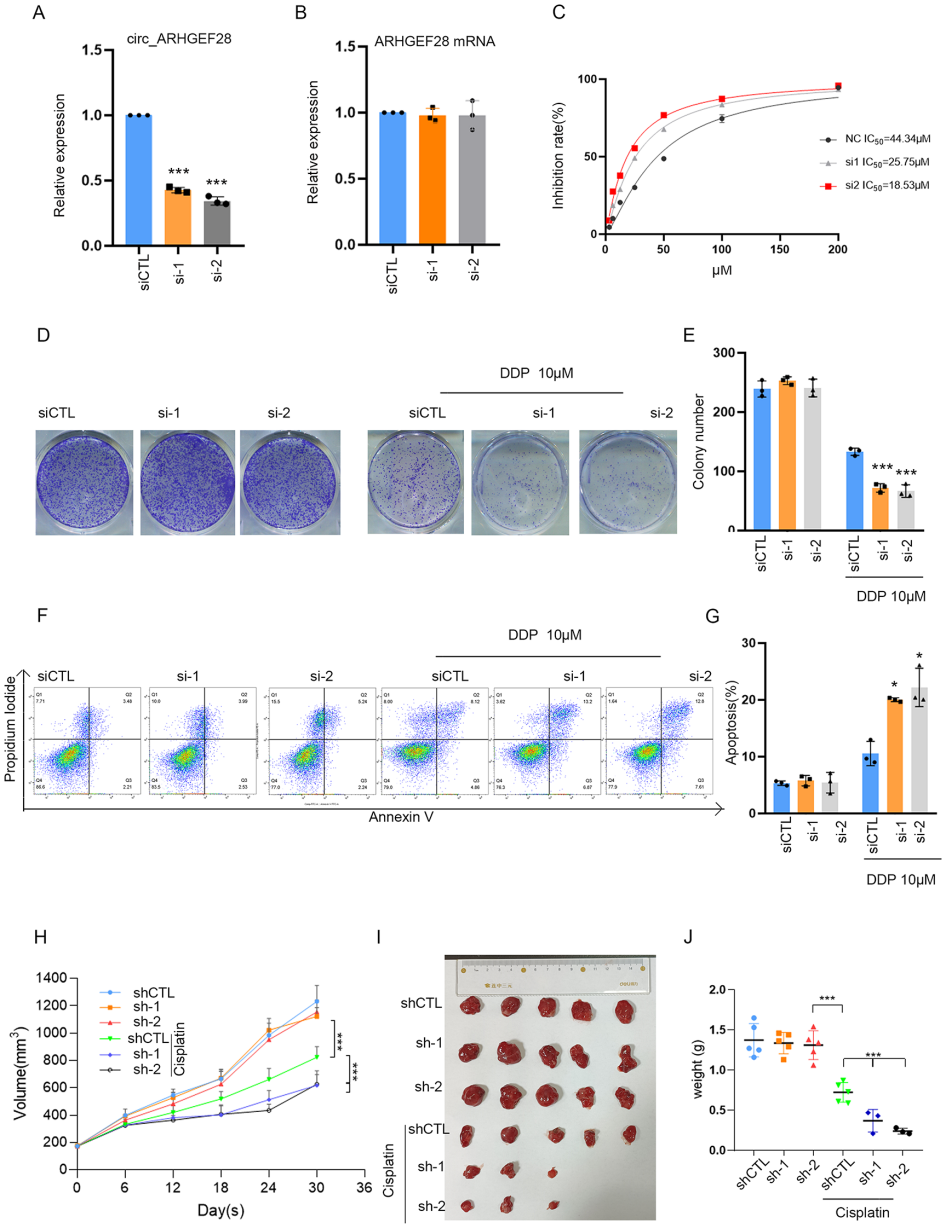
The circ_ARHGEF28 Promotes Cisplatin Resistance in Ovarian Cancer In Vitro and In Vivo. **A** The silencing efficacy of two siRNA against circ_ARHGEF28, as indicated by qPCR. *** means *p* < 0.001. **B** Linear ARHGEF28 mRNA was not changed by silencing circ_ARHGEF28. **C**. Silencing circ_ARHGEF28 decreased the IC50 of A2780R cells to cisplatin. **D**-**E** Silencing circ_ARHGEF28 decreased the cisplatin resistance of A2780R cells, as detected by colony formation assay. *** means *p* < 0.001. **F**-**G** Silencing circ_ARHGEF28 decreased the cisplatin resistance of A2780R cells, as detected by apoptosis assay. * means *p* < 0.05. **H**-**J** Growth curve (**H**) tumor image (**I**) Tumor weights (**J**) of A2780R xenografts treated with cisplatin, A2780R cells transfected with control shRNA (as Vector) or Plko-tet-on-sh circ_ARHGEF28 (as sh circ_ARHGEF28) were injected subcutaneously of nude mice. When tumor size reached ~ 150mm3, mice were treated with cisplatin 4 mg/kg consequently, and fed with doxycycline (doxy) (2 mg/ml) to induce circ_ARHGEF28 (*n* = 5 per group). *** means *p* < 0.001

To elucidate circ_ARHGEF28’s functional role in vitro, gain and loss of function experiments were conducted in A2780R and A2780 cells. Two siRNAs targeting circ_ARHGEF28 achieved over 60% silencing efficiency in A2780R cells (Fig. 3A), while circ_ARHGEF28 expression in A2780 cells was enhanced threefold (Figure S1A). Importantly, the silencing of circ_ARHGEF28 did not alter linear ARHGEF28 mRNA levels (Fig. 3B). Silencing of circ_ARHGEF28 resulted in a reduced half-maximal inhibitory concentration (IC_50_) of A2780R cells to cisplatin (Fig. 3C), whereas overexpression of circ_ARHGEF28 in A2780 cells increased their IC_50_ to cisplatin (Figure S1B). Additionally, colony formation ability and apoptosis rates in A2780R cells were unaffected by circ_ARHGEF28 modulation alone. However, when combined with cisplatin treatment, silencing of circ_ARHGEF28 significantly inhibited colony formation (Fig. 3D-E) and increased apoptosis rates (Fig. 3F-G). Conversely, circ_ARHGEF28 overexpression in A2780 cells led to enhanced colony formation (Figure S1C-D) and reduced apoptosis rates (Figure S1E-F) upon cisplatin treatment.

To assess circ_ARHGEF28’s role in cisplatin resistance in vivo, A2780R cells with a Tet-inducible shRNA-circ_ARHGEF28 system (pLko-Tet-On) were subcutaneously injected into nude mice. Following induction with doxycycline (DOX) and cisplatin treatment (4 mg/kg intraperitoneally daily for one week), a significant reduction in tumor growth and weight was observed, particularly when circ_ARHGEF28 silencing was combined with cisplatin, leading to almost complete tumor regression in some cases (Fig. 3H, I, and J).

### The circ_ARHGEF28 binds to MST1 protein

**Fig. 4:**
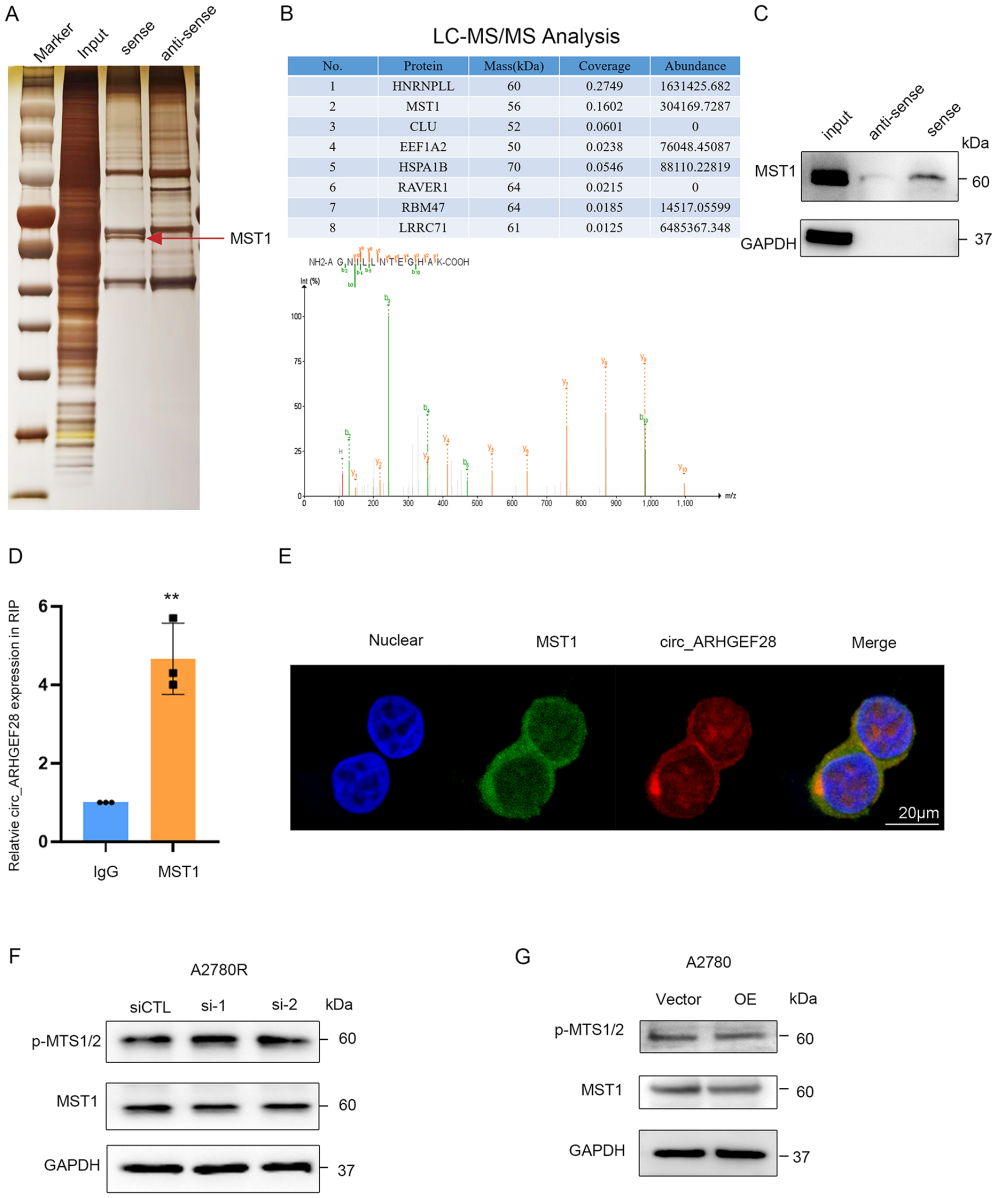
The circ_ARHGEF28 Binds to MST1 Protein. **A** Silver staining of proteins bound to circ_ARHGEF28. The RNA pull-down assay was performed with A2780R cell lysates. A specific band (red arrow) was identified as MTS1 by mass spectrometry. **B** Top list of circ_ARHGEF28 binding protein. **C** MTS1 bound to circ_ARHGEF28 was confirmed by Immunoblotting. **D** The circ_ARHGEF28 bound with MST1 was confirmed by RNA immunoprecipitation (RIP). **E** Colocalization of MST1 and circ_ARHGEF28 in A2780R cells was indicated in immunofluorescence. **F** MST1 expression and phosphorylation were not affected by silencing circ_ARHGEF28 in A2780R cells, as indicated by immunoblotting. **G** MST1 expression and phosphorylation were not affected by overexpressing circ_ARHGEF28 in A2780 cells, as indicated by immunoblotting

The interaction with proteins is a well-known functional mechanism of circRNAs in regulating biological functions. To investigate how circ_ARHGEF28 contributes to cisplatin resistance in ovarian cancer, RNA pull-down assays and mass spectrometry were utilized to predict and identify proteins interacting with circ_ARHGEF28 (Fig. 4A). MST1, a key component of the Hippo signaling pathway known for its tumor-suppressing capabilities, emerged as a top potential binding protein and was identified as a circ_ARHGEF28-binding protein (Fig. 4B). This interaction was further confirmed by RNA pull-down assay, Western blotting (Fig. 4C), and RNA immunoprecipitation (RIP) using an antibody against MST1 in A2780R cells (Fig. 4D).

Immunofluorescence studies further verified the co-localization of MST1 and circ_ARHGEF28, predominantly in the cytoplasm of A2780R cells (Fig. 4E), suggesting a direct binding interaction between circ_ARHGEF28 and MST1 protein.

Contrary to expectations, MST1 expression remained stable, and the phosphorylation of MST1/2 — the active form of MST1 — did not undergo significant changes upon silencing and overexpressing circ_ARHGEF28 (Fig. 4F and G).

These findings indicate that while circ_ARHGEF28 binds and interacts with MST1, it does not influence the stability or phosphorylation status of MST1/2.

### The circ_ARHGEF28’s role in inhibiting MST1/2 dimerization and suppression of the hippo pathway

**Fig. 5:**
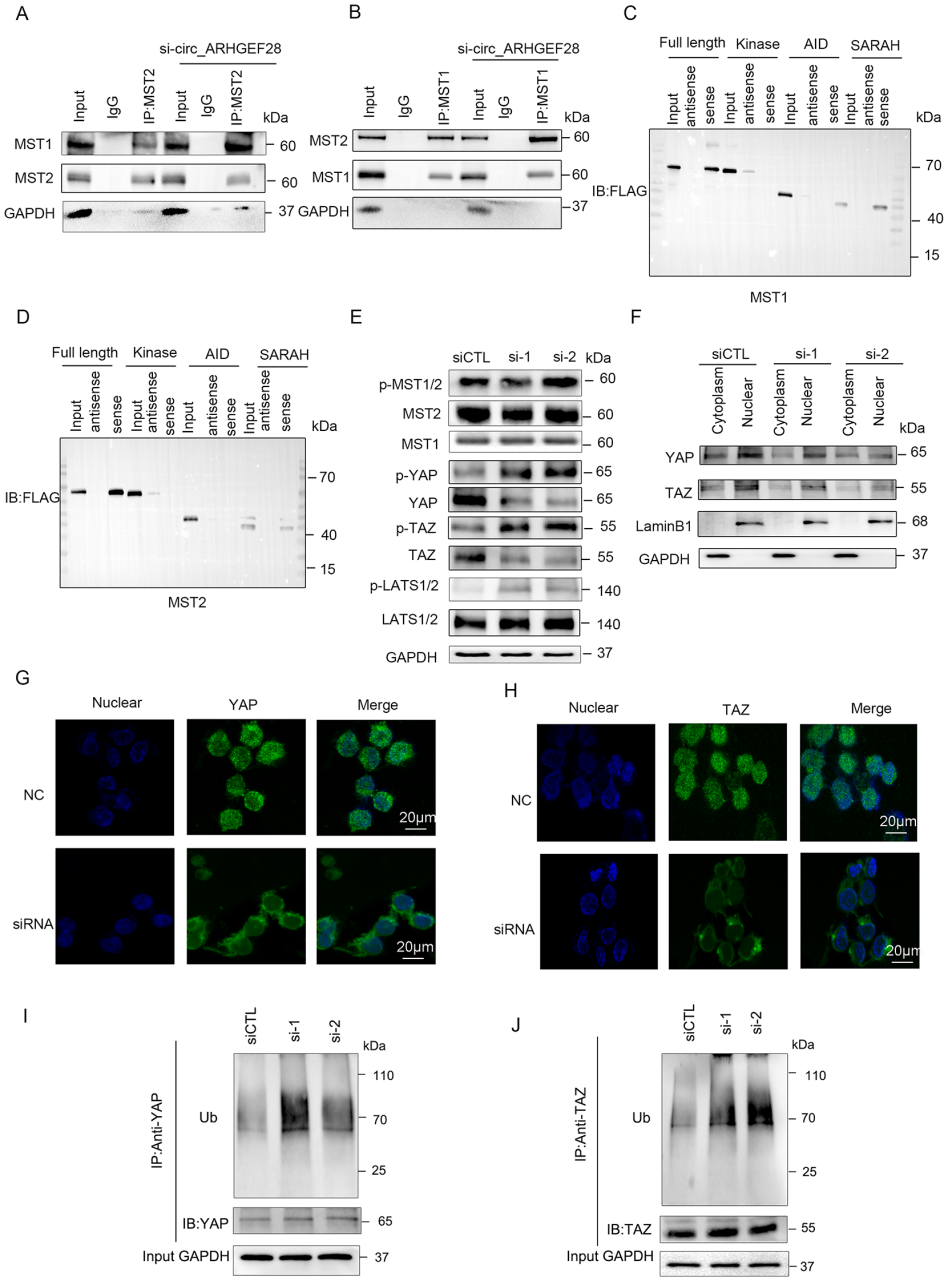
The circ_ARHGEF28’s Role in Inhibiting MST1/2 Dimerization and Suppression of the Hippo Pathway. **A**-**B** The binding between MTS1 and MTS2 was induced by silencing circ_ARHGEF28, as indicated by immunoblotting. **C**.RNA pull-down and immunoblotting showing the interaction of sequentially truncated fraction of MTS1 bound with circ_ARHGEF28. **D** RNA pull-down and immunoblotting showing the interaction of sequentially truncated fraction of MTS2 bound with circ_ARHGEF28. **E** The expression of downstream proteins of MST1/2 in hippo pathway(p-LATS1/2, p-YAP, p-TAZ) was increased when silencing circ_ARHGEF28 in A2780R cells, as indicated by immunoblotting. **F** In A2780R cells, YAP/TAZ higher expressed in the nucleus. Silencing circ_ARHGEF28 reduced the YAP/TAZ expression in the nucleus and cytoplasm. **G**-**H** Silencing circ_ARHGEF28 reduced the YAP/TAZ expression in nucleus and cytoplasm in A2780R cells, especially in the nucleus as indicated by immunofluorescence. **I** Silencing circ_ARHGEF28 increased the ubiquitination of YAP in A2780R cells. **J** Silencing circ_ARHGEF28 increased the ubiquitination of TAZ in A2780R cells

Investigating circ_ARHGEF28’s mechanism in fostering cisplatin resistance, while not altering MST1 stability or phosphorylation, led to exploring its influence on MST1/2 dimerization, a pivotal process in activating the Hippo pathway. Notably, circ_ARHGEF28 silencing resulted in increased binding between MST1 and MST2 (Fig. 5A-B). RNA pull-down and immunoblotting analyses pinpointed the SARAH coiled-coil domains of MST1 and MST2, key to MST1/2 dimerization, as the binding sites for circ_ARHGEF28 (Fig. 5C-D).

Further studies revealed that key downstream effectors of the Hippo pathway, such as phosphorylated LATS1/2, YAP, and TAZ, were upregulated in circ_ARHGEF28-silenced A2780R cells (Fig. 5E and Figure S3A), whereas overexpression of circ_ARHGEF28 in A2780 cells decreased their expression (Figure S2A and Figure S5A). This indicates circ_ARHGEF28’s regulatory role over these downstream elements.

Given the pathway’s functionality, phosphorylated YAP/TAZ typically undergo ubiquitination and degradation, preventing their nuclear translocation, which is associated with tumor progression and resistance to treatment [21, 26]. Intriguingly, total YAP/TAZ expression diminished in circ_ARHGEF28-silenced A2780R cells, with a more pronounced reduction in nuclear expression (Fig. 5F and Figure S3B). Immunofluorescence further illustrated these alterations (Fig. 5G-H). Conversely, circ_ARHGEF28 overexpression in A2780 cells enhanced YAP/TAZ expression both cytoplasmically and nucleically, predominantly nucleically (Figure S2B and Figure S5B).

Addressing the paradox between increased phosphorylated YAP/TAZ and decreased total YAP/TAZ, an analysis of YAP/TAZ ubiquitination was conducted. This revealed increased ubiquitination upon circ_ARHGEF28 silencing in A2780R cells (Fig. 5I-J and Figure S3C-D) and decreased ubiquitination upon its overexpression in A2780 cells (Figure S2C-D and Figure S5C-D).

Thus, the results underscore circ_ARHGEF28’s function in inhibiting MST1/2 dimerization, leading to the suppression of the Hippo pathway. Consequently, this inhibition impedes YAP/TAZ phosphorylation, circumventing their degradation in the cytoplasm, and results in their accumulation and nuclear translocation, ultimately fostering cisplatin resistance in ovarian cancer.

**Fig. 6:**
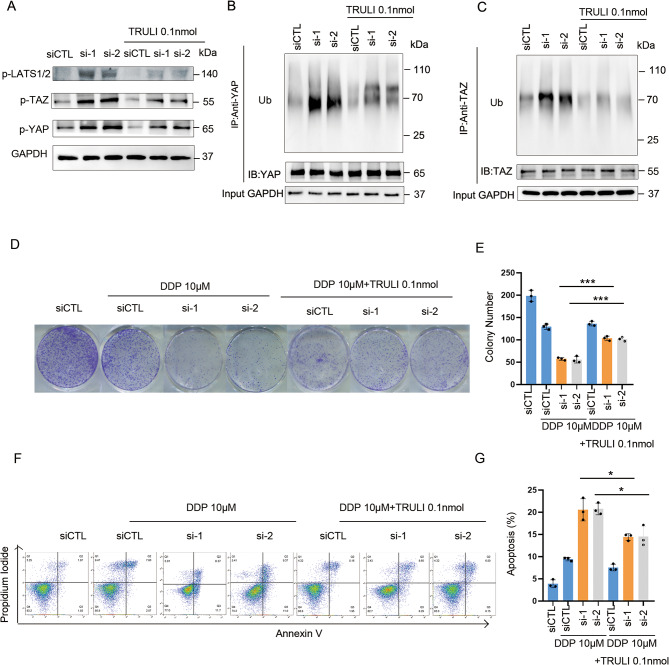
Inhibition of the Hippo Pathway Mitigates Cisplatin Sensitivity Reduction Caused by Silencing circ_ARHGEF28. **A** Addition with LATS1/2 inhibitor TRULI reduces the phosphorylation of LATS1/2, YAP, and TAZ, which is induced by silencing circ_ARHGEF28. **B** Addition with LATS1/2 inhibitor TRULI reduces the ubiquitination of YAP, which is induced by silencing circ_ARHGEF28. **C** Addition with LATS1/2 inhibitor TRULI reduces the ubiquitination of TAZ, which is induced by silencing circ_ARHGEF28. **D**-**E** In addition with LATS1/2 inhibitor TRULI rescued the reduction of colony number induced by silence of circ_ARHGEF28 and cisplatin treatment. *** means *p* < 0.001. **F**-**G** In addition with LATS1/2 inhibitor TRULI rescued the increase of apoptosis induced by silence of circ_ARHGEF28 and cisplatin treatment. * means *p* < 0.05

#### Inhibition of the hippo pathway mitigates cisplatin sensitivity reduction caused by silencing circ_ARHGEF28

To substantiate the hypothesis that circ_ARHGEF28 mediates cisplatin resistance through suppression of the Hippo pathway, the LATS1/2 inhibitor TRULI was employed to counteract the pathway activation resulting from circ_ARHGEF28 silencing. Initial experiments showed that TRULI effectively reduced the expression of phosphorylated LATS1/2 and YAP/TAZ induced by circ_ARHGEF28 silencing (Fig. 6A and Figure S4A). Furthermore, the enhanced ubiquitination of YAP/TAZ observed with circ_ARHGEF28 silencing was mitigated by TRULI treatment (Fig. 6B-C and Figure S4B-C).

Subsequent investigations assessed whether phenotypic changes induced by circ_ARHGEF28 silencing could be reversed by inhibiting the Hippo pathway. It was found that TRULI restored the colony formation capacity (Fig. 6D-E) and reversed the increased apoptosis rates (Fig. 6F-G) brought about by circ_ARHGEF28 silencing.

These results reinforce the notion that circ_ARHGEF28 facilitates cisplatin resistance by suppressing the Hippo pathway.

**Fig. 7:**
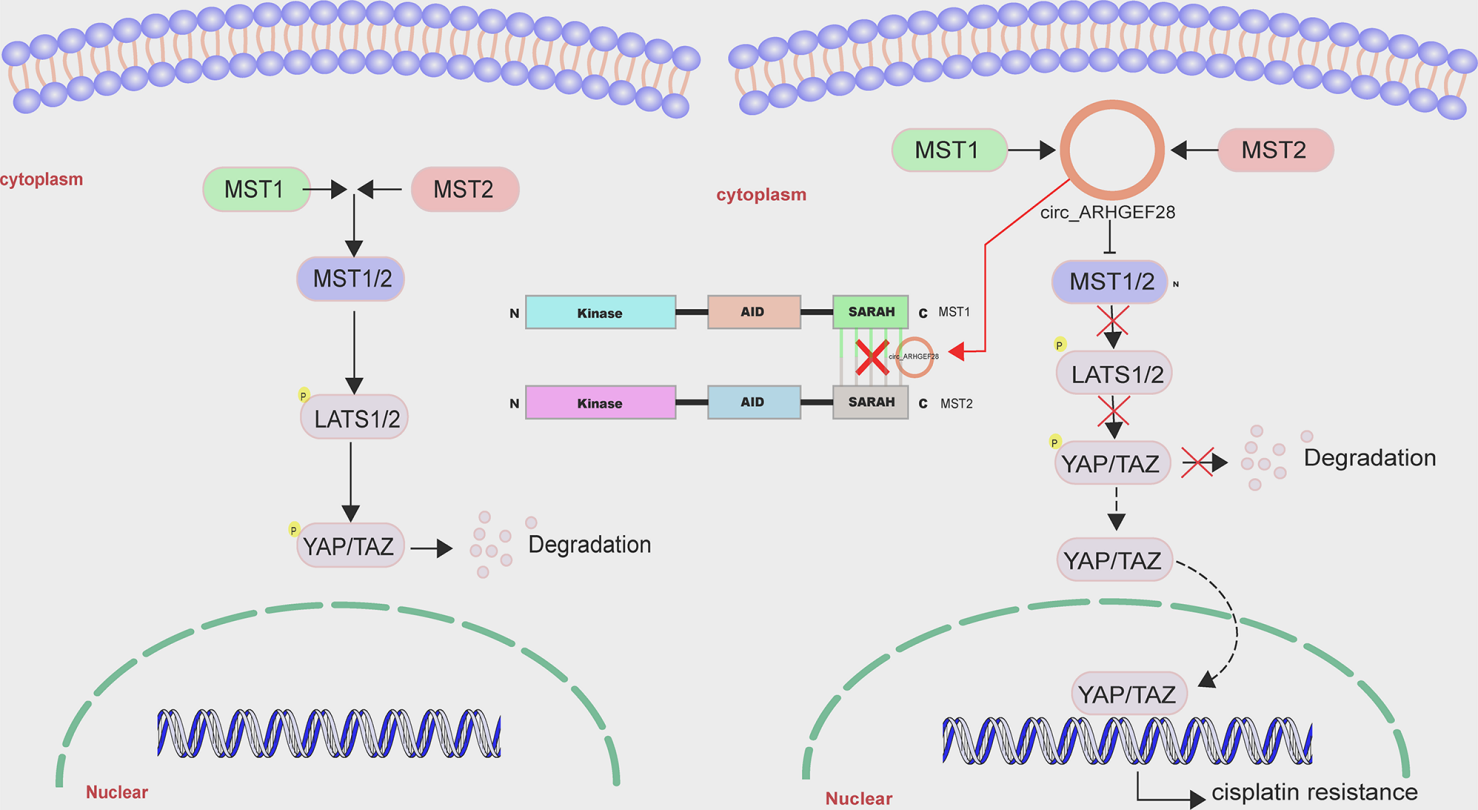
The schematic working model of circ_ARHGEF28 promoting cisplatin resistance in ovarian cancer via suppression of the Hippo pathway. The circ_ARHGEF28 is present to be a factor in blocking the binding SARAH domain of MST1/2 to de-activate the Hippo pathway to prevent the degradation of YAP/TAZ. Further YAP/TAZ accumulated in the cytoplasm and translocated to the nucleus to activate the downstream genes and induced cisplatin resistance in ovarian cancer

In summary, the findings of this study demonstrate that circ_ARHGEF28 directly interacts with MST1/2, obstructing the binding domain of MST1/2, which in turn suppresses the Hippo pathway. This suppression reduces the ubiquitination and degradation of YAP/TAZ, leading to their accumulation and subsequent nuclear translocation, thereby contributing to cisplatin resistance in ovarian cancer (Fig. 7).

## Discussion

The Hippo pathway is instrumental in regulating cellular proliferation, differentiation, and organ size, and is recognized as a tumor suppressor in humans [27, 28]. Central to this pathway are the Mammalian Ste20-like kinases 1/2 (MST1/2), which are homologues of the Hippo (Hpo) gene and pivotal regulators of the Hippo signaling cascade [29]. Mutations or loss of function in the Hippo gene have been linked to organ overgrowth [30], as well as the proliferation and metastasis of tumors, including ovarian cancer [31]. Large tumor suppressor 1/2 (LATS1/2), downstream factors to MST1/2, are typically activated through phosphorylation by MST1/2 (p-MST1/2), although in some instances, LATS1/2 activation occurs independently of p-MST1/2 [32, 33]. In this study, it was discovered that the activation of LATS1/2 was not driven by p-MST1/2, but rather by the dimerization of MST1/2, underscoring the significance of both MST1/2 dimerization and phosphorylation in initiating the Hippo pathway.

Several circRNAs have been identified as contributing to cisplatin resistance in ovarian cancer, with potential roles as biomarkers and therapeutic targets [34, 35]. This study uncovered a novel circRNA, circ_ARHGEF28, linked to cisplatin resistance and poor progression-free survival (PFS) in ovarian cancer. Meanwhile, the ARHGEF28 gene, associated with Rho guanyl-nucleotide exchange factor activity, has been previously reported to correlate with resistance to concurrent chemoradiotherapy in rectal cancer [36]. However, studies focusing on circ_ARHGEF28, derived from the ARHGEF28 gene, are relatively scarce.

This research marks the first instance of identifying a link between circ_ARHGEF28 and cisplatin resistance in ovarian cancer. Diverging from the predominantly studied mechanism of miRNA sponging in circRNA research, circ_ARHGEF28 primarily binds to RNA-binding proteins. It uniquely acts as a protein sequester, shielding the SARAH coiled-coil domains of MST1 and MST2, a mechanism distinct from other reported circRNAs that typically regulate downstream factors like LATS1/2 and YAP/TAZ [37]. By inhibiting the Hippo pathway’s activation, circ_ARHGEF28 contributes to the development of cisplatin resistance. The dimerization of MST1/2 is a crucial step in activating the Hippo pathway and suppressing tumor growth [38]. However, the proliferation and apoptosis appear unaffected by the silencing or overexpression of circ_ARHGEF28, suggesting that circ_ARHGEF28’s expression might be a response to cisplatin exposure and indicative of a “genome evolution” in cancer cells [39].

The suppression of the Hippo pathway leads to the dephosphorylation and nuclear translocation of YAP/TAZ, critical processes in tumor development [40, 41]. This study observed that silencing circ_ARHGEF28 reduced overall YAP/TAZ expression while paradoxically increasing phosphorylated YAP/TAZ levels. The phosphorylated YAP/TAZ in the cytoplasm would be recognized by the proteasome and finally be ubiquitinated and degraded. As a result, when the phosphorylated YAP/TAZ increased with circ_ARHGEF28 silencing, the ubiquitination of YAP/TAZ increased and the total expression of YAP/TAZ decreased. This inconsistency was attributed to heightened ubiquitination and degradation of phosphorylated YAP/TAZ in the cytoplasm.

Additionally, circ_ARHGEF28’s correlation with progression-free survival (PFS) positions it as a potential predictor of platinum sensitivity and therapeutic effectiveness in ovarian cancer. While a higher level of circ_ARHGEF28 was not significantly associated with overall survival (OS), this could be attributed to limited sample sizes and variations in individualized treatment plans, warranting further investigation.

## Conclusion

The study establishes circ_ARHGEF28 as a key factor associated with cisplatin resistance and poorer progression-free survival (PFS) in ovarian cancer. It operates by shielding the SARAH coiled-coil domains of MST1/2, thereby suppressing the Hippo pathway and promoting cisplatin resistance in ovarian cancer. The distinctive expression pattern of circ_ARHGEF28 emerges as a potential biomarker for predicting the response to cisplatin therapy in ovarian cancer. Its role in modulating key signaling pathways underscores the significance of circRNAs in cancer biology and opens avenues for targeted therapeutic strategies.

### Electronic supplementary material

Below is the link to the electronic supplementary material.

Supplementary Material 1

## Data Availability

No datasets were generated or analysed during the current study.

## Abbreviations

circRNA: Circular RNA
IC_50_: Half-maximal inhibitory concentration
MST1: Mammalian STE20-like protein kinase 1
MST2: Mammalian STE20-like protein kinase 2
LATS1: Large tumor suppressor 1
LATS2: Large tumor suppressor 2
YAP: Yes-associated protein
TAZ: Transcriptional co-activator with PDZ-binding motif
PFS: Progression-free survival
OS: Overall survival
FISH: Fluorescence in situ hybridization
