# Supplementary material for: Circular RNA circ_ARHGEF28 inhibits MST1/2 dimerization to suppress Hippo pathway to induce cisplatin resistance in ovarian cancer
Source: Cancer Cell Int. 2024 Jul 21;24:256. doi: 10.1186/s12935-024-03451-w (PMC11264966; doi:10.1186/s12935-024-03451-w)
Supplement: Supplementary file 1 — Supplementary Material 1 [file 12935_2024_3451_MOESM1_ESM.docx]

**Supplementary Table 1** **Correlations of circ_ARHGEF28 Expressing with Clinicopathological Status in 150 Cases of Ovarian Cancer Patients**

| Characteristic |  | All patients [case(%)] |
| --- | --- | --- |
| Total |  | 150 |
| Age |  |  |
| ≤55  >55 |  | 98(65.33)  52(34.67) |
| Histological grade  Middle-high differentiation 3+4  Low differentiation 1+2 |  | 83(55.33)  67(44.67) |
| Histological type  Serous cystadenocarcinoma  Mucinous cystadenocarcinoma  Endometrioid carcinoma  Other  Figo stage  I  II  III  IV  Ascites with tumor cells  Yes  No  Vital status at last follow-up  Alive  Dead  Type of surgery  Primary debulking surgery  Interval debulking surgery |  | 101(67.33)  23(15.33)  15(10.00)  11(7.33)  5(3.30)  12(8.00)  98(65.33)  35(23.33)  87(58.00)  63(42.00)  117(78.00)  33(22.00)  91(60.67)  59(39.33) |

**Supplementary Table 2 QPCR Primers Used in the Manuscript**

| Gene | Forward Sequence | Reverse Sequence |
| --- | --- | --- |
| circ_ARHGEF28 | 5’-AGTTTGGGGTATGTTTTTATTAGTA-3’ | 5’-AAAATAACACTTCCCCTCCCAACCT-3’ |
| Circ_ERPINE2_0001 | 5’-CCCGAGAACACAAAGAAACGC-3’ | 5’-AAGAGGGGGAGATGCCAGTT-3’ |
| Circ_UGGT2_0030 | 5’-ACAGAACTTCCCCATAAAAGCCAG-3’ | 5’-TGGAGCGAGTAGAGTGGATGT-3’ |
| Circ-TEX9-0003 | 5’-GCTATGGGACTATTGCTGTG-3’ | 5’-CTGTTATCGCCATCTACTTG-3’ |
| Circ_SOS2_0052 | 5’-CTTGAAAGACTAGAATCCTTC-3’ | 5’-TTCTTGTCAATGGTCCCTC‐3’ |
| Circ_LDLRAD3_0003 | 5’-GACCAGAGAACCCGGCAG-3’ | 5’-CAGCGTCATGAGGTTGTTCC-3’ |

**Supplementary Table 3. siRNAs in the Manuscript**

| Gene | siRNA Sequence |
| --- | --- |
| Control siRNA | 5’-UUCUCCGAACGUGUCACGU-3’ |
| circ_ARHGEF28 si 1 | 5’-AGAAAAGAAGGUUGGUGAUUU-3’ |
| circ_ARHGEF28 si 2 | 5’-CUAAAGAAAAGAAGGUUGGUG-3’ |

**Supplementary Table 4. Antibodies and Inhibitors Used in the Manuscript**

| **Antibody/Inhibitor** | **Company** | **Cat.** |
| --- | --- | --- |
| MST1 (D8B9Q) Rabbit mAb | Cell Signaling Technology | 14946 |
| MST2 Rabbit Antibody | Cell Signaling Technology | 3952 |
| Anti-MST1/MST2 (Phospho T180 + T183) [EPR1467Y] | Abcam | ab247466 |
| Phospho-LATS1/2(Ser909/Ser872) Antibody | Affinity | AF8163 |
| YAP (D8H1X) XP® Rabbit mAb | Cell Signaling Technology | 14074 |
| Phospho-YAP (Ser127) (D9W2I) Rabbit mAb | Cell Signaling Technology | 13008 |
| TAZ (E9J5A) XP® Rabbit mAb  Phospho-TAZ (Ser89) Antibody | Cell Signaling Technology  Affinity | 72804  AF4315 |
| LATS1/2 inhibitor TRULI | Selleck | E1061 |
